# The NBD-NBD interface is not the sole determinant for transport in ABC transporters

**DOI:** 10.1186/2050-6511-13-S1-A78

**Published:** 2012-09-17

**Authors:** Yaprak Dönmez, Zahida Parveen, Peter Chiba, Thomas Stockner

**Affiliations:** 1 Institute of Pharmacology, Center for Physiology and Pharmacology, Medical University of Vienna, 1090 Vienna, Austria; 2 Institute of Medical Chemistry, Center for Pathobiochemistry and Genetics, Medical University of Vienna, 1090 Vienna, Austria

## Background

The ABC (ATP-binding cassette) transporter superfamily constitutes one of the largest classes of membrane transporters. ABCB1 contains two functional nucleotide binding sites (NBSs) at the interface of the two nucleotide binding domains (NBDs), whereas ABCB11 has one degenerate ATP binding site. According to the structural alignments, ABCB1 and ABCB11 differ by only four residues in the NBD-NBD interface, all of them located at NBS1: E556M, G1178R, Q1180E and S474E. It has been shown that a mutation of the Walker B glutamate (E556) abolishes steady-state ATP hydrolysis and drug transport activities of ABCB1 [1]. We tested the hypothesis that function may be restored in ABCB1 when NBS1 is engineered on the basis of ABCB11.

## Methods

These four residues were mutated in ABCB1 according to ABCB11. Wild-type or mutant ABCB1-transfected cells were used to measure rhodamine 123 transport by flow cytometry. First-order rate constants corresponding to efflux rate were plotted as a function of ABCB1 expression, which was determined by MRK16 staining.

## Results

The E556M mutation of the catalytic glutamate resulted in loss of transport function. While the double mutation in the LSGGQ motif (G1178R, Q1180E) reduced transport to below 20%, no measurable rhodamine 123 efflux was observed in either the triple mutant (E556M, G1178R, Q1180E) or the quadruple mutant (E556M, G1178R, Q1180E, S474E).

## Conclusions

Engineering of ABCB1 NBS1 to mimic ABCB11 NBS1 yields a non-functional transporter, indicating that the NBD-NBD interface is not the sole determinant for transport in ABC exporters.
